# First report of ‘*Candidatus* Anaplasma camelii’ and high molecular prevalence of *Anaplasma marginale* in dromedary camels (*Camelus dromedarius*) from Somalia

**DOI:** 10.1007/s11250-026-04885-2

**Published:** 2026-02-20

**Authors:** Aamir M. Osman, Abdalla M. Ibrahim, Ahmed A. Hassan-Kadle, Marcos R. André, Flávia C. M. Collere, Anna C. B. Mongruel, Daniel Lee, Caroline Tostes Secato, Thállitha S. W. J. Vieira, Rosangela Z. Machado, Rafael F. C. Vieira

**Affiliations:** 1 Graduate Program on Veterinary Sciences, Universidade Federal do Paraná, Curitiba, Paraná Brazil; 2 Somali One Health Centre, Abrar Research and Training Centre, Mogadishu, Somalia; 3 Department of Animal Health and Veterinary Services, Ministry of Livestock, Forestry, and Range, Mogadishu, Somalia; 4 Abrar Research and Training Centre, Mogadishu, Somalia; 5 Vector-Borne Bioagents Laboratory (VBBL), Department of Pathology, Reproduction and One Health, Faculty of Agrarian and Veterinary Sciences, São Paulo State University (FCAV/UNESP), Jaboticabal, Brazil; 6 Department of Chemistry, The University of North Carolina at Charlotte, Charlotte, NC USA; 7 Center for Computational Intelligence to Predict Health and Environmental Risks (CIPHER), The University of North Carolina at Charlotte, Charlotte, NC USA; 8 Department of Epidemiology and Community Health, The University of North Carolina at Charlotte, Charlotte, NC USA

**Keywords:** *'Candidatus* Anaplasma camelii’, *Anaplasma marginale*, Tick-borne diseases, Dromedary camels, Sub-Saharan Africa

## Abstract

Anaplasmosis, a zoonotic disease caused by *Anaplasma* species, threatens Somalia’s dromedary population, crucial to the nation’s economy and society. This study examines its occurrence in dromedaries from two Somali bioclimatic regions using molecular and serological methods. A total of 155 blood and serum samples from dromedaries in Benadir and Lower Shabelle were analyzed. Serum samples underwent rMSP5-based ELISA for anti-*Anaplasma marginale* IgG antibodies. DNA was extracted from dromedary blood samples followed by molecular screening included nested PCR for the 16S rRNA gene of *Anaplasma* spp. and qPCR for the *msp1β* gene of *A. marginale*. Positive samples underwent further PCR and sequencing targeting five additional genes for definitive species confirmation and phylogenetic analysis. Of 155 dromedaries tested, 30 (19.4%) were seropositive for *Anaplasma marginale* by iELISA, and 117 (75.5%) were qPCR-positive for the *msp1β* gene. Notably, 97 animals that were seronegative by iELISA tested positive by qPCR, and the overall agreement between the two tests was substantial (κ = 0.62). Among 22 qPCR-positive samples, semi-nested PCR (snPCR) targeting the *msp1α* gene detected six positives (27.3%). The molecular prevalence of *A. marginale* was significantly higher in Benadir (84.6%) compared to Lower Shabelle (56.9%). A significant association was also observed between *A. marginale* infection and tick infestation, with infested dromedary camels showing lower odds of being positive (OR = 0.4). Initial nPCR screening identified *Anaplasma* spp. positive in 45 (29%) of animals, with 23 (14.8%) showing co-infections between *A. marginale* and *Anaplasma* spp. Further analysis of 25 selected nPCR-positive samples revealed positivity for ITS 17 (68%), *groEL* 12 (48%), and *gltA* 15 (60%) genes, though *msp4* was not detected. Phylogenetic analysis of 16S rRNA sequences showed clustering with ‘*Candidatus* Anaplasma camelii’ from Saudi Arabian dromedaries. This is the first report of ‘*Ca.* A. camelii’ and *A. marginale* in dromedary from Somalia, highlighting the need for further research on its impact on animal health.

**Supplementary Information:**

The online version contains supplementary material available at https://doi.org/10.1007/s11250-026-04885-2.

## Introduction

Anaplasmosis is an emerging zoonotic disease caused by *Anaplasma* species, which are obligate intracellular, Gram-negative bacteria belonging to the family Anaplasmataceae and the order Rickettsiales. This order was reclassified in 2001 to include several genera, namely *Anaplasma*, *Ehrlichia*, *Neorickettsia*, and *Wolbachia* (Dumler et al. 2001; Parola et al. 2009). Currently, there are eight recognized *Anaplasma* species: *A. marginale*, *A. centrale*, *A. ovis*, *A. bovis*, *A. platys*, *A. phagocytophilum* (Rar and Golovljova 2011; Dumler et al. 2001), *A. odocoilei* (Tate et al. 2013), and *A. capra* (Li et al. 2015a). All these infect livestock, with *A. platys, A. phagocytophilum,* and *A. capra* identified as zoonotic bacteria (Battilani et al. 2017; Li et al. 2015a). In dromedaries, infections with *A. marginale*, *A. platys*, *A. ovis*, *A. phagocytophilum*, an *A. platys* genetically related strain, and ‘*Candidatus* Anaplasma camelii’, have been reported across various regions. Specifically, *A. platys*, *A. ovis*, *A. phagocytophilum*, and an *A. platys* genetically related strain were reported in Iran (Bahrami et al. 2018; Rassouli et al. 2020; Noaman 2018), *A. marginale* in Egypt (Mahmoud et al. 2023), and *A. ovis*, and *A. phagocytophilum* in Tunisia (BenSaid et al. 2014; Selmi et al. 2019). An *A. platys* genetically related strain was reported in dromedaries from Algeria (Bessas et al. 2022) and Kenya (Getange et al. 2021), while *A. platys* and its genetically related strain were detected in Nigeria (Onyiche et al. 2020) and China (Li et al. 2015b). In Saudi Arabia, *A. platys* genetically related strain and *A. phagocytophilum* were also reported in dromedaries (Bastos et al. 2015; Al-Nabati et al. 2022).

In the biological cycles of *Anaplasma* species, both invertebrate and vertebrate hosts play distinct roles, with ticks serving as the primary vectors for transmission (Aubry and Geale 2011). Hard ticks (Acari: Ixodidae), such as *Rhipicephalus* spp., *Dermacentor* spp., and *Ixodes ricinus*, are the main vectors. In Somalia, ticks of the genera *Rhipicephalus, Hyalomma*, and *Amblyomma* commonly infest camels and other livestock. The most frequently reported species on Somali dromedaries include *Rhipicephalus pulchellus, R. humeralis, Hyalomma dromedarii, H. rufipes, H. marginatum,* and *Amblyomma lepidum* (Collere et al. 2025). These ticks are well adapted to arid and semi-arid pastoral landscapes, feed on multiple host species, and may facilitate cross-species transmission of pathogens. Additionally, other hematophagous arthropods, including horseflies (*Tabanus* spp.) and stable flies (*Stomoxys calcitrans*), may mechanically transmit *A. marginale* (Selmi et al. 2022; Kocan et al. 2010, 2004). *Anaplasma* can also spread through other means, including blood sucking dipterans, contaminated objects such as needles and ear tags, castration equipment, and transplacental transmission, depending on the species involved (Aubry and Geale 2011).

The epidemiology of anaplasmosis is complex due to the diversity of *Anaplasma* spp., the wide range of host species, and the role of vectors in transmission. These bacteria typically cycle asymptomatically between enzootic ticks and wild or domestic vertebrate hosts; however, certain species, such as *A. marginale*, can cause severe wasting and anemia when transmitted to its natural hosts, such as cattle (Kocan et al. 2010). In dromedaries, natural infections with *A. marginale* have been associated with clinical signs, including fever, anorexia, anemia, jaundice, dullness, diarrhea, emaciation, lacrimation, abortion, and infertility (El-Alfy et al. 2024; Ismael et al. 2016). Depending on the species involved, *Anaplasma* shows specific tropisms for different blood cells, such as erythrocytes, monocytes, macrophages, granulocytes, or platelets (Silaghi et al. 2017). The presence of endosymbiotic bacteria in ticks further complicates detection, mechanisms of development, pathogenicity, and the clinical manifestation of these infections in livestock (Gofton et al. 2015). Endosymbionts can modulate the pathogenicity of related bacteria by influencing host physiology, and their concentration in tick tissues, such as salivary glands, suggesting a role in pathogen transmission. Moreover, dominance can overshadow the presence of less abundant pathogens, making it difficult to detect them during profiling (Gofton et al. 2015; Ahantarig et al. 2013). Animals that recover from acute infection often remain lifelong carriers, acting as reservoirs of infection for naïve livestock populations and potentially triggering endemic outbreaks or epizootics (Mahmoud et al. 2023).

Over 60% of the world’s dromedaries are kept by pastoralists in the Greater Horn of Africa (GHA), which includes Djibouti, Ethiopia, Kenya, Somalia, South Sudan, Sudan, and Uganda. Somalia hosts nearly 9.1 million dromedaries, making it one of the largest populations worldwide. Dromedaries hold significant economic value in Somalia, providing food, acting as a currency, and serving as essential transport for resources like milk and water, while also symbolizing social status (Osman 2024)). These animals are uniquely adapted to survive and thrive in Somalia’s arid and semi-arid climates, with most dromedaries managed by nomadic pastoralists. However, these pastoral practices increase their vulnerability to tick-borne diseases (TBDs). Despite the global significance of dromedaries, they remain neglected and understudied, especially regarding emerging tick-borne bacterial infections. In recent decades, several new and re-emerging tick-borne rickettsial pathogens of public and veterinary health concern have emerged (Makgabo et al. 2023; Li et al. 2015a). In Africa and the Middle East, the newly detected’*Ca.* Anaplasma camelii’ has been reported in dromedaries from Kenya (Bargul et al. 2021; Getange et al. 2021), Morocco (Ait Lbacha et al. 2017), Tunisia (Selmi et al. 2019), Saudi Arabia (Bastos et al. 2015), and the United Arab Emirates (Ishag et al. 2024). Despite the vulnerability of dromedaries, the prevalence of *Anaplasma* spp. in Somalia remains unknown. To address this gap, our study aimed to investigate the occurrence of *Anaplasma* species in dromedaries across two distinct bioclimatic regions in Somalia, using molecular and serological techniques.

## Material and methods

### Study area and design

**Fig. 1:**
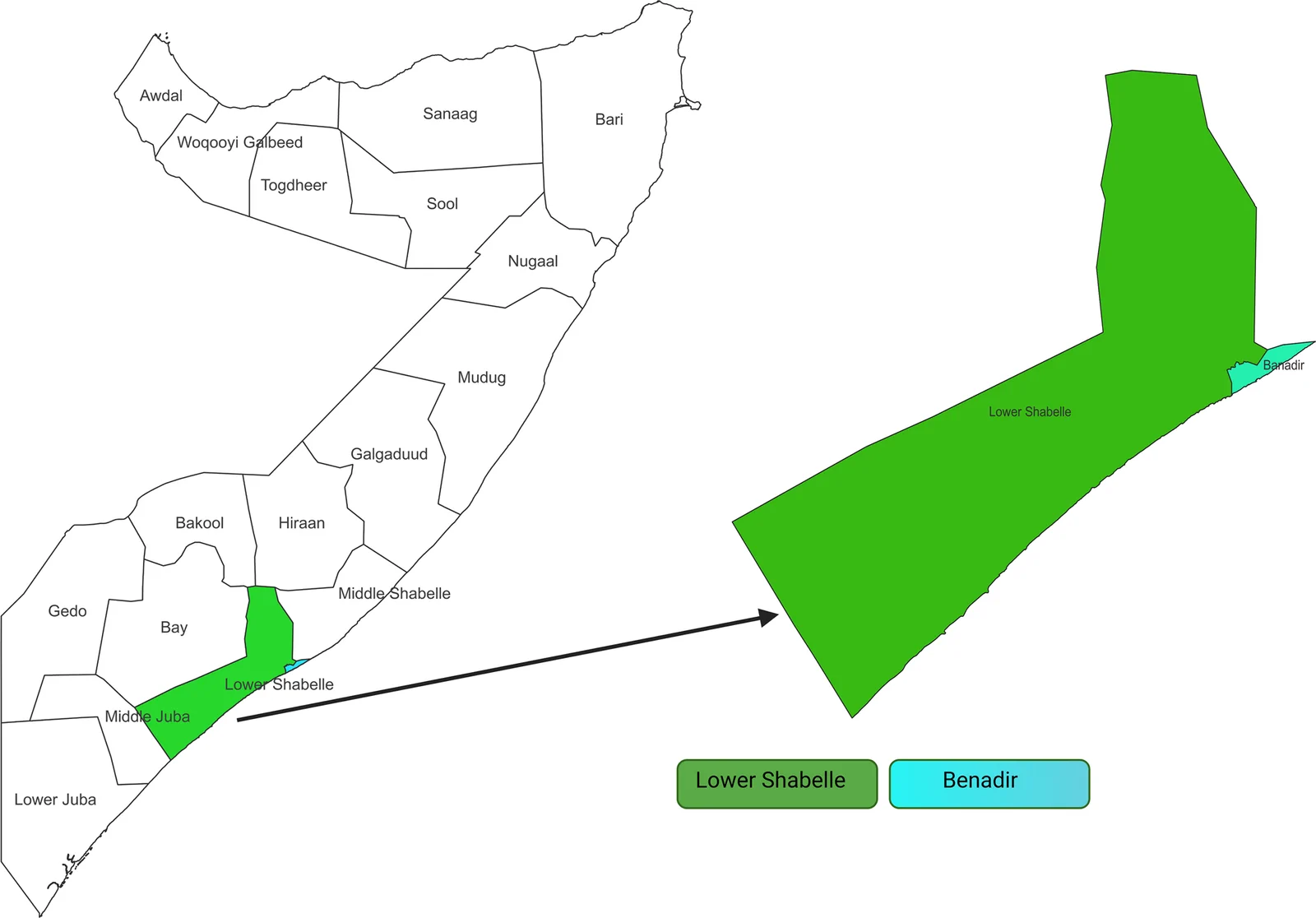
Map of Somalia showing the sampling sites. The highlighted Celeste (Benadir region) and green (Lower Shabelle region) indicate the locations of the sampled area. The figure was generated and modified using QGIS software version 3.26.0

A cross-sectional study was conducted between December 2018 and March 2022 in the Benadir (2.1065° N, 45.3933° E) and Lower Shabelle (1.8670° N, 44.5502° E) regions of Somalia (Fig. 1). A convenience sampling strategy was employed. A total of 155 dromedaries (104 from Lower Shabelle and 51 from Benadir) were sampled once during this period; no animal was resampled. Herds were selected based on accessibility and farmer consent, and within herds, animals were selected with the cooperation of the owners. All dromedary camels belonged to pastoralist production systems, the predominant husbandry practice in Somalia, which relies on seasonal migration for pasture and water. A total of 155 dromedary blood samples from previous studies were retrieved and used (Collere et al. 2025; Osman et al. 2024).

### Detection of IgG antibodies against *A. marginale* rMSP5

Dromedary serum samples were tested for anti-*A. marginale* IgG antibodies using a commercial rMSP5-based indirect enzyme-linked immunosorbent assay (iELISA) (Imunodot Diagnostics^®^, Jaboticabal, São Paulo, Brazil), as previously described (Machado et al. 1997). Briefly, ELISA plates (Maxisorp^®^; Nunc, Thermo Scientific, Brazil) containing the rMSP5 at a concentration of 2.5 μg/mL in 0.05 M sodium carbonate-bicarbonate buffer (pH 9.6) were incubated in a moist chamber at 37 °C for 90 min. After three washes with PBS-Tween 20 buffer (pH 7.2), positive and negative reference sera, along with the diluted test serum samples (1:100 in PBS-Tween 20 solution with 5% normal rabbit serum), were added to the plates. The plates were then incubated again at 37 °C for another 90 min. Following three additional washes with PBS-Tween 20, dromedary camels conjugated IgG (KPL SureBlue™, SeraCare Life Sciences, Milford, USA) was added at a dilution of 1:1,000 in PBS-Tween 20 with 5% normal rabbit serum, followed by incubation and washing. Subsequently, the substrate for alkaline phosphatase, P-nitrophenyl phosphate (Sigma^®^, St. Louis, MO, USA), diluted to 1 mg/mL in diethanolamine buffer (pH 9.8; Sigma^®^, St. Louis, USA), was added. The ELISA plates were sealed with aluminum foil and incubated at room temperature for 30 min. Absorbance readings were taken using Dynex MRX TC Microplate Reader (Dynex Technologies, Shirley, New York, USA) with a 405 nm filter. Negative control sera were collected from newborn calves prior to colostrum ingestion and confirmed *A. marginale*-negative by qPCR and serology. Positive control sera were obtained from naturally infected cattle verified as *A. marginale*-positive through qPCR and serology. The cutoff values were determined as 2.5 times the mean absorbance of the negative control sera (Machado et al. 1997).

### DNA extraction

DNA was extracted from dried blood spots (DBS) on filter paper (FTA^®^ card, Whatman^®^, GE Healthcare, Buckinghamshire, United Kingdom) and from 200 μL of whole blood using a commercial kit (IndiMag^®^ Pathogen Kit, Qiagen for Indical Bioscience, Leipzig, Germany), according to the manufacturer’s instructions. For DBS, three discs were removed from each sample using a 3 mm Punch (Harris Uni-Core™, Qiagen, Hilden, Germany) and placed into clean RNase/DNase-free 1.5 mL microtubes. Pre-treatment involved adding 200 μL of phosphate-buffered saline (PBS) to the macerated FTA discs, which were then heated at 96 °C for 10 min. Filter paper discs without blood served as negative controls to monitor cross-contamination. DNA from EDTA-blood samples was extracted using the same commercial kit. Ultra-pure water was used as a negative control in each extraction batch to ensure no cross-contamination occurred. DNA samples were stored at −20 °C until PCR assays were performed.

### Polymerase chain reaction (PCR) assays for *A. marginale*

Conventional PCR (PCR) for the mammalian endogenous gene glyceraldehyde-3-phosphate dehydrogenase (*gapdh*) was performed in all samples to monitor DNA extraction (Birkenheuer et al. 2003). Following this, DNA samples were screened by a quantitative real-time PCR (qPCR) assay targeting the *msp1β* gene of *A. marginale*, as described elsewhere (Carelli et al. 2007). Each sample was tested in duplicates, with a final reaction volume of 10 μL. The reaction mixture comprised 1 μL of the DNA sample, 0.2 μM of the hydrolysis probe, 0.9 μM of each primer (Table 1), 5 μL of PCR buffer (GoTaq Probe qPCR Master Mix, Promega^®^, Madison, Wisconsin, USA), and sterilized ultrapure water (Nuclease-Free Water, Promega^®^, Madison, Wisconsin, USA). Amplification reactions were performed using low-profile multiplate plates in a QuantStudio 5 system thermal cycler (Thermo Fisher Scientific^®^, Waltham, Massachusetts, USA). The standard curve was calibrated using serial dilutions of a gBlock™ (Integrated DNA Technologies, Coralville, IA, USA). All parameters were analyzed according to the standards established by the MIQE guidelines (Minimum Information for Publication of Quantitative Real-Time PCR Experiments) (Bustin et al. 2009).

**Table 1:** Description of primers, amplicon sizes and thermal conditions used in PCR assays for *Anaplasma* spp.

Gene	Target species	Aim	Primer	Sequence (5′-3′)	Annealing temperature (°C)	Base pair (pb)	Reference
*msp1β*	*Anaplasma marginale*	Screening	AM-F:	TTGGCAAGGCAGCAGCTT	60 ◦C	~95	Carelli et al. (2007)
			AM-R:	TTCCGCGAGCATGTTGCAT			
			AM-probe:	6FAM-5′-TCGGTCTAACATCTCCAGGCTTTCAT-3′-BHQ1			
*msp1α*	*Anaplasma marginale*	Characterization	msp1αF^1^	GTG CTT ATG GCA GAC ATT TCC	57◦C^1^	~643	Castañeda-Ortiz et al. (2015)
			msp1αR^1,2^	CTC AAC ACT CGC AAC CTT GG			
			msp1αNF^2^	CGC ATT ACA CGT TCC GTA TG	65◦C^2^		
*msp* 4	*Anaplasma* spp.	Characterization	MSP45	GGGAGCTCCTATGAATTACAGAGAATTGTTTAC	68◦C	~870	De La Fuente et al. (2004)
			MSP43	CCGGATCCTTAGCTGAACAGGAATCTTGC			
Short 16S rRNA gene	*Anaplasma* spp.	Screening	gE3a^1^	CACATGCAAGTCGAACGGATTATTC	55 ◦C^1,2^	~932	Massung et al. (1998)
			gE10R^1^	TTCCGTTAAGAAGGATCTAATCTCC			
			gE2^2^	GGCAGTATTAAAAGCAGCTCCAGG		~546	
			gE9f^2^	AACGGATTATTCTTTATAGCTTGCT			
Large 16S rRNA gene	*Anaplasma* spp.	Characterization	AE1-F	AAGCTT AAC ACA TGC AAG TCG AA	59 ◦C	~1,406	Oh et al. (2014)
			AE1-R	AGT CAC TGA CCC AAC CTT AAA TG			
(ITS—23S–5S)	*Anaplasma* spp.	Characterization	ITS2F	AGGATCTGACTCTAGTAACGAG	58 ◦C	~300	Rejmanek et al. (2012)
			ITS2R	CTCCCATGTCTTAAGACAAAG			
*gltA*	*Anaplasma* spp.	Characterization	F4b	CCGGGTTTTATGTCTACTGC	55 ◦C^1,2^	∼800	Müller et al. (2018)
			R1b	CGATGACCAAAACCCAT			
			HER-5136F	TTYATGTCYACTGCTGCKTG		∼650	
			EHR-778 R	GCNCCMCCATGMGCTGG			
*groEL*	*Anaplasma* spp.	Characterization	groEL-1F	ATAGCTAGCATAATTACCCAGAGC	55 ◦C^1,2^	∼339	Zhang et al. (2016)
			groEL-1 R	GGTTAGTTCTGCTTTCGATGC			
			groEL-2F	TTATGTCTATGCGCCGTG			
			groEL-2 R	CGGACCTTGCCACATTTT			

Samples that tested positive for *A. marginale* by qPCR with Cq values ranging from 16.3 to 34.7 were further analyzed using PCR assays with genus-specific primers targeting the *msp4* gene of *A. marginale* and *A. ovis*, as previously described (De La Fuente et al. 2004). Additionally, a snPCR assay was performed to amplify the *msp1α* gene of *A. marginale*, as described elsewhere (Castañeda-Ortiz et al. 2015). The cycling conditions and primers used in these PCR assays are detailed in Table 1. A cattle blood DNA sample experimentally infected with *A. marginale* (Jaboticabal strain) served as the positive control, while sterile ultrapure water (Nuclease-Free Water, Promega Corporation, Madison, WI, USA) was used as the negative control in all analyses.

### PCR assays for *Anaplasma* spp.

All dromedary DNA samples were initially screened for *Anaplasma* spp. using a nested PCR (nPCR) targeting the 16S rRNA gene, as previously described (Massung et al. 1998). Twenty-five selected positive samples which have strong bands on electrophoresis from this screening assay were subjected to additional PCR assays for molecular characterization using various molecular markers, including large 16S rRNA (Oh et al. 2014), *gltA* (Müller et al. 2018), and *groEL* (Zhang et al. 2016), and ITS 23S-5S intergenic region (Rejmanek et al. 2012) genes. The cycling conditions and primer sequences used in these assays are provided in Table 1. All PCR reactions were carried out in a total final volume of 25 μL, with 5 μL of DNA used for conventional PCR and 1 μL from the first reaction in the nPCR assay. The reaction mixture included 1.25 U of Go Hot Taq DNA Polymerase (Promega^®^, Madison, Wisconsin, USA), PCR buffer (10×, 100 mM Tris-HCl, pH 9.0, 500 mM KCl), 0.2 mM of each deoxynucleotide (dATP, dTTP, dCTP, and dGTP) (Invitrogen^®^, Carlsbad, CA, USA), 1.5 mM Magnesium Chloride (Promega^®^, Madison, Wisconsin, USA), 0.5 μM of each primer (Invitrogen^®^, Carlsbad, CA, USA), and sterile ultrapure water (Promega^®^, Madison, Wisconsin, USA). DNA from *A. phagocytophilum* was used as the positive control, while ultrapure sterile water (Promega^®^, Madison, Wisconsin, USA) served as the negative control in all PCR assays.

### PCR cloning procedures

Two *msp1α* of *A. marginale* and four large 16S rRNA (1,406 bp) gene of *Anaplasma* spp. amplicons were cloned using the pGEM T-easy vector (Promega^®^, Madison, Wisconsin, USA) as per the manufacturer’s instructions. The ligation reaction products were used to transform One Shot™ Mach1™ T1R chemically competent *Escherichia coli* cells (Invitrogen, Cat # C8620–03), with a transformation efficiency of 10^9^–10^10^ CFU/ng DNA, as previously described (Furquim et al. 2021). Plates were prepared using LB (Luria Bertani medium - Tryptone; Yeast Extract; NaCl; distilled water q.s.p. [ThermoFisher Scientific, Waltham, MA, USA]) agar medium cointaning 100 μg/mL ampicillin, 40 μL X-gal (5-bromo-4-chloro-3-indolyl-β-Dgalactoside; 0.026%) and 20 μL IPTG (isopropylthio-β-galactoside; 0.82 mM), allowing the selection of clones based on the blue/white colony screening system. Plasmid DNA was extracted using the Wizard^®^ Plus SV Minipreps DNA Purification System (Promega^®^, Madison, WI, USA) according to the manufacturer’s recommendations, and the samples were stored at −20 °C for later use in PCR to confirm successful cloning. PCR-confirmed colonies containing the target gene fragment underwent plasmid DNA extraction and were submitted to Sanger sequencing using the primers M13-F (5′ CGCCAGGGTTTTCCCAGTCACGAC-3′) and M13-R (5′ GTCATAGCTGTTTCCTGTGTGA-3′) (Lau et al. 2010).

### Electrophoresis, amplicon purification and Sanger sequencing

The amplified PCR products were separated via electrophoresis on 1% agarose gels, which were stained with ethidium bromide (Life Technologies™, Carlsbad, CA, USA). Electrophoresis was performed at 90 V for 50 min. Following separation, the gels were exposed to ultraviolet light using a ChemiDoc MP Imaging System (BioRad^®^), and images were captured using Image Lab Software version 4.1. Selected positive samples were purified using the Promega Wizard^®^ PCR and Gel Clean-Up system (Promega^®^, Madison, WI, USA) and sequenced in both directions using the same forward and reverse PCR primers by Sanger sequencing (Sanger et al. 1977). Purified DNA was quantified using a NanoDrop 2000c spectrophotometer. The obtained sequences were compared with those available in the GenBank^®^ database by the BLASTn tool (National Center for Biotechnology Information, Bethesda, MD, USA) (Altschul et al. 1990). The gene sequences obtained herein were submitted to GenBank^®^ database under the following accession numbers: for 16S rRNA gene short fragment (PQ479628 - PQ479633), and for 16S rRNA gene extended fragment (PQ482489 - PQ482492).

### Phylogenetic and distance analyses

A Maximum Likelihood phylogram was constructed using the 16S rRNA (1,406 bp) sequences of *Anaplasma* spp., incorporating sequences obtained in this study alongside homologue sequences available at the GenBank^®^ database (http://www.ncbi.nlm.nih.gov/genbank). The 16S rRNA (1,406 bp) gene was chosen for phylogenetic analysis due to its ability to provide superior resolution compared to partial sequences, as it includes essential regions for generating robust and accurate phylogenies. Geneious Prime v.2023.0.4 software was utilized to ensure the quality of electropherograms and to assemble consensus sequences. These consensus sequences were then subjected to BLASTn analysis (National Center for Biotechnology Information, Bethesda, MD, USA) (Altschul et al. 1990) to identify similarities with sequences deposited in the GenBank^®^ database. Multiple sequence alignment with selected sequences from GenBank^®^ was performed using MAFFT online software (https://mafft.cbrc.jp/alignment/server/). The best-fit model of nucleotide substitution, determined using jModeltest v0.2.1.10 (Darriba et al. 2012) based on the Akaike Information Criterion (AIC), was GTR + G. The resulting phylogram was visualized using FigTree software version 1.4.4. Pairwise distance calculations were performed using an alignment of three extended sequences of the *Anaplasma* 16S rRNA gene obtained in the present study and 16 homologue sequences retrieved from GenBank^®^ (OQ909470, OQ909469, OQ909468, OQ909467, OQ909466, M60313, KX765882, KF843828, KF843827, KF843825, KF843824, EU439943, AY262124, AB211164, AB211164, AB196721) using the *p*-distance model, incorporating both transition and transversion substitutions with uniform rates. The analysis was conducted with MEGAX software (Kumar et al. 2018; Stecher et al. 2020). The resulting pairwise distance matrix was transferred to a Microsoft Excel 2016 spreadsheet, where distances were transformed in percentage values and a heat map was generated.

### Statistical analysis

Data analyses were performed with SPSS Statistics software^®^ (IBM Corp, Armonk, NY, USA, version 26). The chi-square test was used to evaluate significant differences between infection/exposure rate and presence of ticks. Odds ratio (OR), 95% confidence intervals (95% CI), and P-values were calculated separately for each variable, and results were considered significant when *p* ≤ 0.05. Data were compiled and analyzed in Epi Info™ software, version 7.2.3.1 (Centers for Disease Control and Prevention, CDC, USA). The kappa coefficient (κ) of agreement among the iELISA and the *msp1β* qPCR assay for *A. marginale* were calculated using a freely available software (http://www.openepi.com). The magnitude of κ coefficients was interpreted as follows: ≤0 poor, 0.01–0.2 slight, 0.21–0.4 fair, 0.41–0.6 moderate, 0.61–0.8 substantial, and 0.81–1 almost perfect agreement (Viera and Garrett 2005).

## Results

### Serology of *A. marginale* rMSP5

Anti *A. marginale* rMSP5-based IgG antibodies were detected in 30/155 (19.4%; 95% CI: 13.45 - 26.5%) dromedaries, being (13/51, 25.5%, 95% CI: 14.3–39.6%) in Lower Shabelle and 17/104 (16.3%, 95% CI: 9.8 - 24.9%) at Benadir region.

### qPCR detection of* A. marginale *and associated factors

The *gapdh* gene was successfully amplified from all dromedary DNA samples. By the *msp1β* gene-based qPCR, 117/155 (75.5%; 95% CI: 67.9–82.0%) (range of Cq values = 10.6 - 37.8) animals tested positive for *A. marginale*. The qPCR performance showed an average efficiency of 97.3%, with an r^2^ of 0.998, a slope of −3.378, and a y-intercept of 40.198. The highest prevalence for *A. marginale* was found in the Benadir region 88/104 (84.6%, 95% CI: 76.2–90.9%), followed by Lower Shabelle region 29/51 (56.9%, 95% CI: 42.2–70.7%). Notably, 97 animals that tested seronegative by the iELISA tested positive for *A. marginale* via the *msp1β* gene-based qPCR. Five dromedaries that tested seropositive by the iELISA tested negative by qPCR. Additionally, 20 (12.9%) animals tested positive and 29 animals (18.7%) tested negative in both assays. There was substantial agreement between the iELISA and the *msp1β* gene-based qPCR assay (k = 0.62) for detecting *A. marginale*. Among the 117 *A. marginale*-positive animals, 52 were infested by ticks. A significant association was found between *A. marginale* positivity and the presence of ticks (OR = 0.4; 95% CI: 0.2–0.8; χ^2^ = 6.6, *p* = 0.01). 79 out of 155 dromedaries (50.9%; 95% CI: 42.8–59.1%) were parasitized by ticks, with an average of 4.4 ticks per animal. The identified tick species included *Rhipicephalus pulchellus* (50.3%), *Hyalomma dromedarii* (29.8%), *Hyalomma rufipes* (10.1%), *Hyalomma marginatum* (4.6%), *Rhipicephalus humeralis* (4.0%), *Amblyomma lepidum* (0.6%), *Amblyomma gemma* (0.3%), and *Ornithodoros* sp. (0.5%) (Collere et al. 2025).

A total of 22 *A. marginale* qPCR-positive samples with Cq values ranging from 16.3 to 34.7 were selected for species characterization. Out of these, all tested negative for the *msp4* gene while six/22 (27.3%; 95% CI: 10.7–50.2%) tested positive for the *msp1α* gene. Unfortunately, multiple attempts to clone the *msp1α* gene for genotyping have failed.

### Nested PCR detection of *Anaplasma* spp.

A total of 45/155 (29.0%, 95% CI: 22.0 - 36.9) dromedaries tested positive for *Anaplasma* spp. by the nPCR based on the 16S rRNA gene, being 36/51 (70.6%, 95% CI: 56.2–82.5%) at the Lower Shabelle and nine/104 (8.7%, 95% CI: 4.0–15.8%) at the Benadir region. Twenty-three (14.8%, 95% CI: 9.6–21.4%) animals tested positive for both *A. marginale* and *Anaplasma* spp. Eight 16S rRNA gene amplicons of *Anaplasma* spp. were sequenced and showed 99.25–100% identity with ‘*Ca.* A. camelii’ detected in dromedaries from Iran and Saudi Arabia (GenBank^®^ KX765882, KF843825, respectively), 100% identity with *A. platys* (GenBank^®^ MN630836) detected from China, 99.4% identity with *A. phagocytophilum* detected in dromedary from Tunisia (GenBank^®^ KC455366), and 98.45% identity with an uncultured *Anaplasma* spp. found in a domestic dog from Brazil (GenBank^®^ MT229120).

From 25 selected *Anaplasma*-positive samples by 16S rRNA gene based on their strong electrophoresis band intensity, 17 (68%) tested positive for the ITS 23S-5S intergenic region, 12 (48%) tested positive for the *groEL* gene, and 15 (60%) were positive for the *gltA* gene. The sequencing of the ITS 23S-5S intergenic region, *groEL*, and *gltA* gene regions yielded poor results, potentially due to factors such as partial DNA degradation, the presence of inhibitors, or challenges specific to these gene regions that affected the sequencing outcomes.

### Phylogenetic analysis and p-distance matrix

**Fig. 2:**
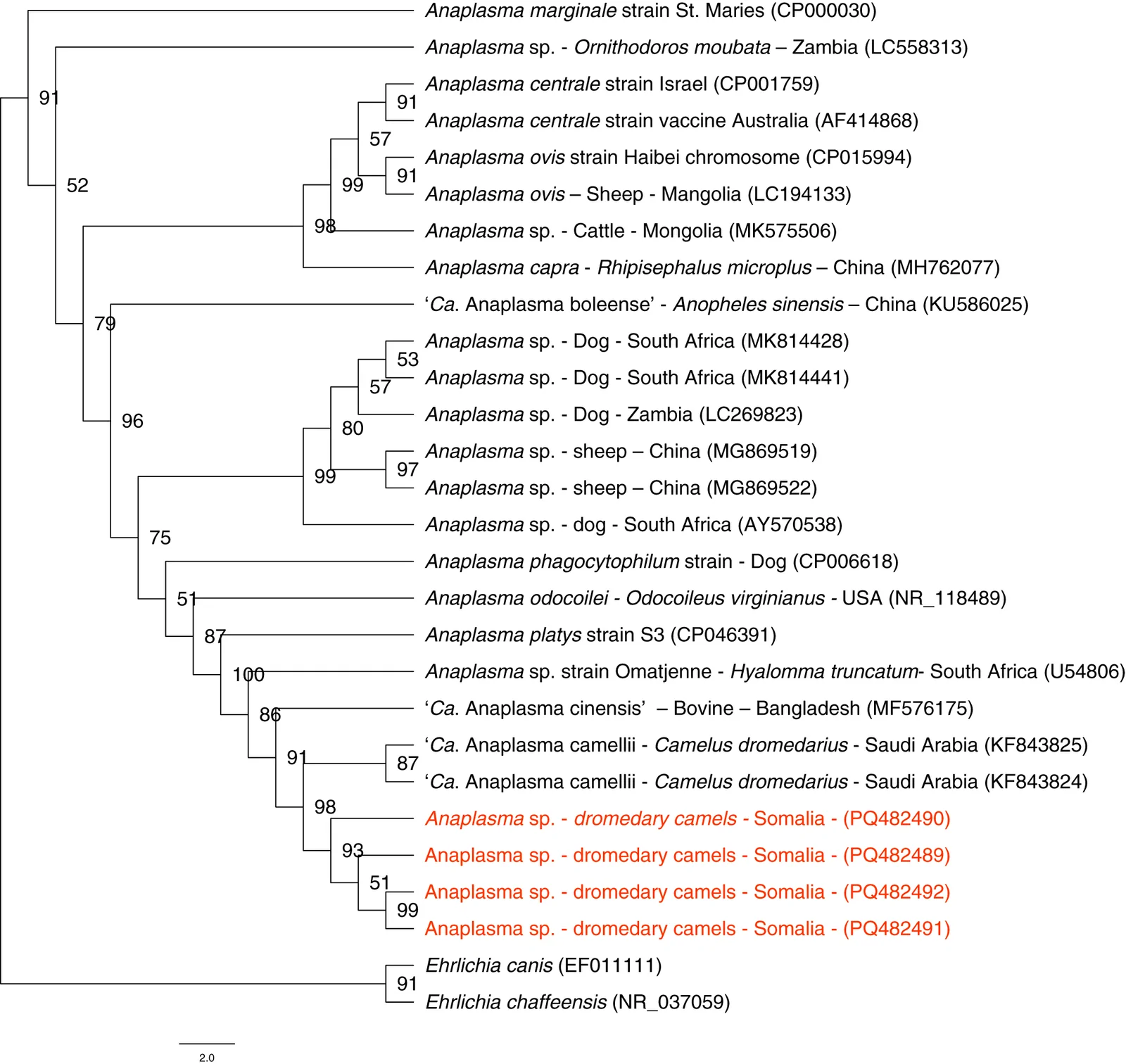
Maximum likelihood phylogrambased on an alignment of 1406 bp of *Anaplasma* spp. 16S rRNA gene, using Maximum likelihood method and GTR+G as evolutionary model. Sequences from the present study were highlighted in red. *Ehrlichia canis* and *E. chaffeensis* were used as an outgroup

Phylogenetic analysis of the 16S rRNA gene of *Anaplasma* spp., using an alignment with total size of 1106 bp, was performed with four sequences detected in the present study and selected sequences of *Anaplasma* spp. available at GenBank^®^ database confirmed the BLASTn analysis. The obtained sequences from the present study grouped with *’Ca.* A. camelii’ sequences detected in dromedary from Saudi Arabia (Fig. 2).

Regarding the *p*-matrix generated herein, the genetic divergence between sequences obtained in the present study and those closely related and retrieved from GenBank^®^ database ranged from 0.00% to 4.63%. Divergence rates among the four sequences obtained in the present study ranged from 0.00% to 0.09%. Comparisons with *A. platys* showed divergence ranging from 0.18% to 0.27%, while comparisons with ‘*Ca* A. camelii’ ranged from 0.00% to 0.18%. The *p*-distance matrix is presented in Supplementary File 1.

## Discussion

This study provides the first molecular and serological evidence of *Anaplasma* spp. infection in dromedary camels in Somalia, addressing a major knowledge gap in a country that hosts one of the largest camel populations globally. The detection of both *A. marginale* and ‘*Ca.* Anaplasma camelii’ highlights the circulation of multiple *Anaplasma* lineages in Somali dromedaries and underscores the potential epidemiological role of camels within pastoral production systems.Our findings revealed a seroprevalence for *A. marginale* rMSP5-based IgG antibodies of 19.4%. Previous studies on *A. marginale* in dromedary camels from Iraq reported lower detection rates, with only 13 out of 120 dromedary camels (10.83%) testing positive using a competitive iELISA based on a recombinant immunodominant antigen (Al-Gharban and Al-Taee 2016). However, considering that the rMSP5 protein used in this study is highly conserved across *Anaplasma* species, the possibility of serological cross-reactivity cannot be ruled out (Strik et al. 2007).

In this study, the molecular prevalence of *A. marginale* based on the *msp1β* gene was significantly higher, with 75.5% of dromedary camels testing positive. Further analysis of positive samples using PCR targeting the *msp4* gene yielded negative results, while snPCR targeting the *msp1α* gene detected 27.3% positive samples. This discrepancy may be explained by the high Cq values observed, indicating that the bacterial load in these samples was low. Such low levels of bacteremia could fall below the detection thresholds of the PCR assays employed. Previous studies on *A. marginale* in Arabian dromedary camels have reported varying prevalence rates. For instance, a study conducted in Saudi Arabia found 44.99% (616/1374) of dromedary camels to be positive for *A. marginale* using PCR targeting the 16S rRNA and *cox1* genes (Al-Nabati et al. 2022). Similarly, a study in Egypt reported a 30% (75/250) prevalence by nPCR targeting the *groEL* gene (Mahmoud et al. 2023). The differences in prevalence rates between these studies and our findings may reflect ecological and husbandry factors unique to Somali pastoral systems. The high density of camels, pervasive tick infestations by genera *Hyalomma*, *Amblyomma*, and *Rhipicephalus* (Collere et al. 2025), and limited use of acaricides could drive intense enzootic transmission, potentially establishing camels as significant reservoirs in the region. The dominant tick genera found on Somali dromedaries are known competent vectors for *Anaplasma* spp. elsewhere. Our findings suggest they are likely the primary drivers of the high transmission cycle observed, creating a self-sustaining camel-tick-camel cycle. Additionally, variations in diagnostic methods likely contributed to variation in reported prevalence. The use of highly sensitive qPCR and snPCR targeting the *msp1β* and *msp1α* genes, respectively, while other studies used different molecular markers, namely 16S rRNA, *cox1*, and *groEL*, which may influence detection sensitivity.

In epidemiological studies, serology is crucial for detecting antibodies, while combining it with molecular diagnostics enhances accuracy. Herein, the overall seroprevalence of *A. marginale* rMSP5-based IgG antibodies was 19.4%, while 75.5% of animals tested positive for *A. marginale* by qPCR targeting the *msp1β* gene. This difference, where the number of positives by qPCR was higher than by ELISA, reflects a pattern also observed in studies involving cattle from Mozambique, which reported an 86.3% seroprevalence by iELISA and a 97.3% positivity rate by qPCR (Fernandes et al. 2019). Specifically, in our study, 97 animals were seronegative by iELISA but qPCR-positive, likely due to the acute phase of infection when antibody levels are below the detection threshold. Conversely, five seropositive animals were qPCR-negative, possibly indicating persistent infections with low bacterial loads or residual antibodies from previously cleared infections. Beyond infection stage, other plausible explanations for this discrepancy should also be considered. Persistence of bacterial DNA after infection clearance, together with the high sensitivity of the qPCR assay, may have contributed to the detection of very low-level bacteremia (Carelli et al. 2007). Indeed, the assay employed a hydrolysis probe and demonstrated excellent repeatability across duplicates, supporting its reliability. On the other hand, the negative qPCR results in a subset of seropositive animals may be attributable to bacterial loads below the detection threshold, as typically seen in chronic *A. marginale* carriers (Kocan et al. 2010). Another possibility is suboptimal performance of the bovine-validated rMSP5 iELISA in dromedary camels, where reduced sensitivity or specificity could arise from imperfect antibody affinity or cross-reactivity. Taken together, these findings highlight the importance of combining serological and molecular tools to achieve a more comprehensive understanding of pathogen occurrence and transmission. Nevertheless, future studies should include multiple molecular targets and further validation of serological assays in camels to strengthen diagnostic reliability and comparability.

Different *Anaplasma* species have been molecularly identified in camels worldwide, including *A. platys*, *A. ovis*, *A. phagocytophilum*, and a genetically related strain of *A. platys* in Iran (Bahrami et al. 2018; Rassouli et al. 2020; Noaman 2018), *A. marginale* in Egypt (Mahmoud et al. 2023), and *A. marginale*, *A. platys*, *A. ovis*, *A. phagocytophilum*, *A. bovis*, and *A. centrale* in Tunisia (BenSaid et al. 2014; Selmi et al. 2019). In Algeria (Bessas et al. 2022) and Kenya (Getange et al. 2021), a genetically related strain of *A. platys* has been reported, while *A. platys* and its genetically related strain were detected in Nigeria (Onyiche et al. 2020) and China (Li et al. 2015b). In Saudi Arabia, *A. platys* genetically related strains and *A. phagocytophilum* were identified (Selmi et al. 2022; El-Alfy et al. 2024). However, it remains unclear whether camels serve as reservoir hosts for any *Anaplasma* species. The detection of *Anaplasma* DNA in camels’ blood could result from exposure to the bacterium through close contact with ruminants that would favor bites from ticks harboring the pathogen. In our study, 29.0% of animals tested positive for *Anaplasma* spp. by nPCR targeting the 16S rRNA gene. Sanger sequencing of eight positive samples revealed high identity with *‘Ca* A. camelii’. Our findings differ from a previous study conducted in the United Arab Emirates (UAE), which detected Anaplasmataceae agents in 12.2% (35/287) of samples, with all sequenced samples corresponding to *‘Ca.* A. camelii’ (Ishag et al. 2024). Additionally, a previous study in Tunisia found a 35% (144/412) prevalence of Anaplasmataceae agents in camels, with sequencing revealing strains closely related to *A. platys*-like (Selmi et al. 2019). This contrast in prevalence and species identification highlights regional differences and suggests the need for further research on the epidemiology of *Anaplasma* spp. in dromedaries.

*‘Candidatus* Anaplasma camelii’, first identified by Bastos et al. (2015) in dromedary camels from Saudi Arabia, is closely related to *A. platys*. While earlier studies have documented its prevalence in camels from diverse regions, 40.3% in Nigeria (Onyiche et al. 2020), 35.85% in Morocco (Ait Lbacha et al. 2017), 17.7% in Tunisia (Belkahia et al. 2015), 78.7% in Kenya (Getange et al. 2021), and 25.7% in the UAE (Ishag et al. 2024), this study further contributes to the expanding geographical understanding of *‘Ca.* A. camelii’ infection in dromedaries. Notably, the detection of *’Ca.* A. camelii’ in deer (*Rusa timorensis*) and cattle from Malaysia (Koh et al. 2018) further highlights the potential for multiple animal hosts to be involved in its transmission, though its pathogenicity remains unclear. Moreover, *’Ca.* A. camelii’ has been detected in ticks from genera *Hyalomma, Amblyomma,* and *Rhipicephalus* in Nigeria (Onyiche et al. 2020) and Kenya (Getange et al. 2021). Given the high prevalence of *Hyalomma, Amblyomma,* and *Rhipicephalus* ticks parasitizing dromedaries in Somalia (Collere et al. 2025), there is a significant possibility that these vectors may play a role in the transmission dynamics within the region. In light of this, it is imperative to investigate the potential role of dromedaries as reservoirs and to explore the implications of ‘*Ca. A. camelii’* in both dromedaries and other susceptible species in Somalia.

When comparing the three extended *Anaplasma* 16S rRNA fragments from dromedaries detected herein with homologous sequences using the *p*-distance matrix, the dromedary sequences exhibited low divergence values (0.00%–0.09%) with sequences previously detected in wild animals including elephant (*Loxodonta africana),* leopard (*Panthera pardus),* impala (*Aepyceros melampus),* and lions (*Panthera leo)* in South Africa. In addition, comparisons with sequences from *‘Ca.* A. camelii’ from Saudi Arabia and Iran showed divergence values ranging from 0.00% to 0.18%. Given the high conservation of this gene and the possibility of > 98.7% similarity between distinct *Anaplasma* species based on 16S rRNA fragments (Caudill and Brayton 2022), further analyses, based on distinct molecular markers, are required to determine whether our sequences represent genotypes closely related to those found in wildlife or camels from other regions.

This study provides crucial initial insights into anaplasmosis in Somali dromedaries, though several limitations should be considered when interpreting the results. First, the study relied on convenience sampling including previously collected serum and blood samples, geographically restricted to two regions, may affect the generalizability of the prevalence estimates to the broader Somali dromedary population. Second, samples were collected during specific time periods rather than across all seasons, potentially introducing seasonal bias, given that tick abundance and transmission intensity of tick-borne pathogens can vary substantially with rainfall patterns and temperature. Furthermore, the phylogenetic characterization of *’Ca*. A. camelii’ relied substantially on a single locus (16S rRNA), and the partial sequencing failures of other gene targets (*groEL, gltA, ITS*) limited a more comprehensive genomic analysis. For *A. marginale*, although *msp1α* gene were successfully amplified in selected samples, direct sequencing could not be performed due to limited amplicon quantity and unsuccessful cloning attempts. Cloning was pursued because *msp1α* contains highly variable and repetitive regions that can yield ambiguous chromatograms in mixed infections; cloning allows separation of variants for accurate strain genotyping and phylogenetic analysis. Finally, while appropriate for this initial descriptive study, the use of univariable statistics means that the influence of potential confounders such as age, sex, and precise tick burden could not be assessed. Despite these constraints, this research provides crucial foundational data by confirming for the first time the presence and circulation of *A. marginale* and *’Ca*. A. camelii’ in Somali dromedaries. Future studies with larger, randomized samples and more comprehensive individual animal metadata will be valuable to perform multivariable analyses and strengthen the generalizability of these important findings.

In conclusion, our study sheds light on the occurrence and molecular characterization of *Anaplasma* spp. in dromedaries in Somalia, representing the first report of *Anaplasma* spp. using both serological and molecular methods. The detection of *’Ca.* A. camelii’ and *A. marginale* confirms their circulation in Somali camel populations and highlights dromedaries as potential participants in local transmission cycles. However, the role of camels as reservoir hosts cannot be inferred from these data alone and requires targeted transmission and vector competence studies. Our findings underscore the need for combining molecular diagnostics with serological methods to achieve a more comprehensive understanding of *Anaplasma* infections and inform effective control strategies.

## supplementary material

Below is the link to the electronic supplementary material.


**Supplementary File 1:** Pairwise distance calculations were performed using an alignment of three extended sequences of the Anaplasma 16S rRNA gene obtained in this study, along with 16 homologous sequences retrieved from GenBank^®^. The analysis was conducted in MEGA X using the p-distance model.


## Data Availability

Partial nucleotide sequencing data from this study are available in GenBank under the following accession numbers: for 16S rRNA gene short fragment (PQ479628 - PQ479633), and for 16S rRNA gene extended fragment (PQ482489 - PQ482492). Additional datasets generated and analyzed are provided as a supplementary file.
